# Supplementary material for: Developing inhibitory peptides against SARS-CoV-2 envelope protein
Source: PLoS Biol. 2024 Mar 14;22(3):e3002522. doi: 10.1371/journal.pbio.3002522 (PMC10939250; doi:10.1371/journal.pbio.3002522)
Supplement: S1 Fig — Following the global proteomics results shown in Fig 2A, the expression of the gene transcripts was examined using qPCR. The expressions of HSPA6 (A), DAGLB (B), IP6K2 (C), AGAP3 (D), and RELB transcripts (E) significantly increased in HEK 293S cells transfected to 2E-mKate2 compared to mock. The expression of the other genes, TNC (F), CALU (G), PKLR (H), NOLC1 (I), and ATF3 (J), did not significantly increase in the transfected HEK 293S cells though all the gene transcriptions slightly increased. Unpaired Student’s t test was used (*** P < 0.001; ** P < 0.01; * P < 0.05; n.s., not significant, n = 6). The data underlying this figure can be found in S1 Data. All the graphs in the figure are mean ± SD. (PDF) [file pbio.3002522.s001.pdf]

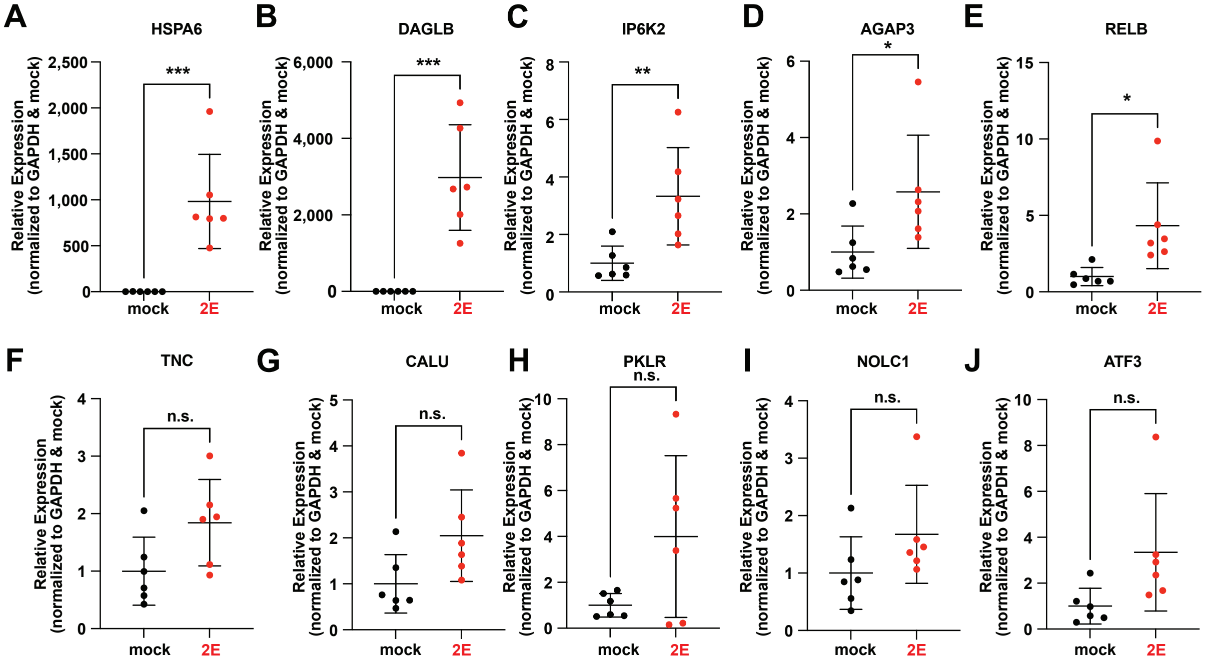

S1 Fig | The effect of SARS2-E overexpression on mammalian transcripts. Following the global proteomics results shown in Fig 2A, the expression of the gene transcripts was examined using qPCR. The expressions of HSPA6 (**A**), DAGLB (**B**), IP6K2 (**C**), AGAP3 (**D**) and RELB transcripts (**E**) significantly increased in HEK 293S cells transfected to 2E-mKate2 compared to mock. The expression of the other genes, TNC (**F**), CALU (**G**), PKLR (**H**), NOLC1 (**I**) and ATF3 (**J**), did not significantly increase in the transfected HEK 293S cells though all the gene transcriptions slightly increased. Unpaired Student's *t*-test was used (*** $P < 0.001$; ** $P < 0.01$; * $P < 0.05$; n.s., not significant, n=6). The data underlying this figure can be found in S1 Data. All the graphs in the figure are mean ± s.d.
