# Supplementary material for: Developing inhibitory peptides against SARS-CoV-2 envelope protein
Source: PLoS Biol. 2024 Mar 14;22(3):e3002522. doi: 10.1371/journal.pbio.3002522 (PMC10939250; doi:10.1371/journal.pbio.3002522)
Supplement: S2 Fig — (A) Relative fluorescent intensity of DND-189 dye in NIH 3T3 cells transfected using mock (n = 32) or 2E-mKate2 plasmid without (-, n = 27) and with TAT-MY18-2ED peptide 24-h incubation (10 μM, n = 50). One-way ANOVA with Tukey’s multiple comparisons test (**** P < 0.0001; n.s. not significant). (B) Representative immunoblot images of HEK 293T cells transfected with SARS2-E fused with YFP (2E-YFP). Anti-SARS2-E (2E-N, clone, N2A5E8, left), GFP (right), and GAPDH antibodies (as loading control, bottom) were used. The rat monoclonal antibody (2E-N mAb, clone N2A5E8) was produced using MY18 peptide conjugated to keyhole limpet haemocyanin (KLH) as the antigen. (C) ELISA assay for the comparison of binding capacity of the anti-SARS2-E monoclonal antibody to wild-type (WT) and 2ED (EE7-8DD) mutant TAT-MY18 peptides. (D) The stability test of iPep-SARS2-E (TAT-MY18-2ED) using the same ELISA assay with anti-SARS2-E antibody. The peptide was incubated at 37°C in phosphate-buffered solution (PBS). (E–G) The toxicity test of iPep-SARS2-E (TAT-MY18-2ED, 10 μM, 48 h) using Jurkat cells and flowcytometry with apoptosis/necrosis assay. Healthy cells (E, %), apoptotic cells (F), and dead/necrotic cells (G) were counted. Camptothecin (10 μM, 3 h) was used as a positive control. One-way ANOVA with Dunnett’s multiple comparisons test was used (**** P < 0.0001; n.s. not significant, compared to non-treated). (H) Immunoprecipitation of 2E protein using Ni column and HEK 293T cells transfected using 2E-YFP with 6xHis-MY18-2ED (2ED) or 6xHis-MY18 wild-type constructs (WT). Anti-GFP antibody was used to blot 2E-YFP protein bands. The data underlying this figure can be found in S1 Data. All the graphs in the figure, except S2C Fig, are mean ± SD. S2C Fig uses single-sample datasets. (PDF) [file pbio.3002522.s002.pdf]

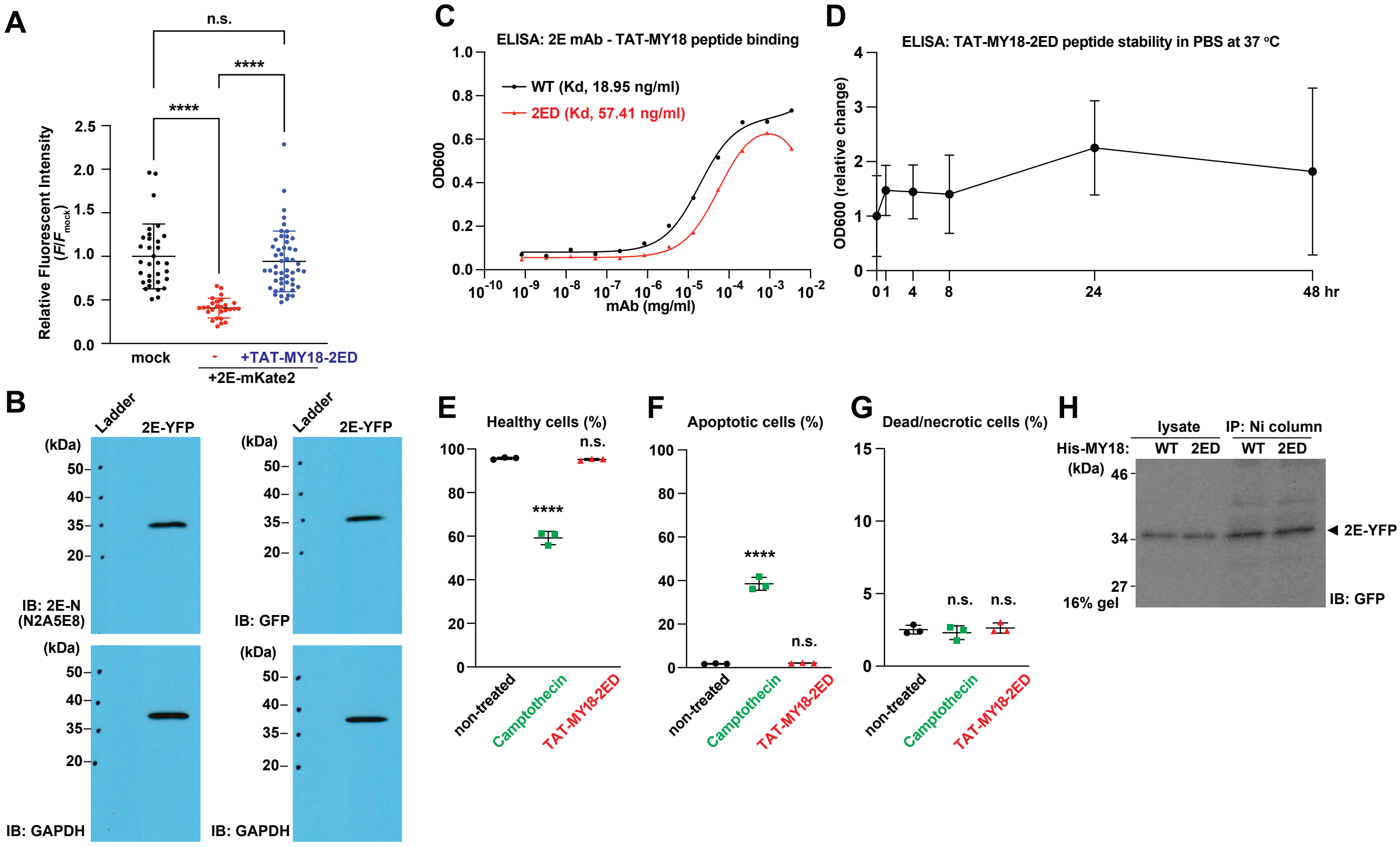

S2 Fig | Characterization of iPep-SARS2-E *in situ* and *in vitro*. (**A**) Relative fluorescent intensity of DND-189 dye in NIH 3T3 cells transfected using mock (*n*=32) or 2E-mKate2 plasmid without (-, *n*=27) and with TAT-MY18-2ED peptide 24-hr incubation (10μM, *n*=50). One-way ANOVA with Tukey's multiple comparisons test (**** $P < 0.0001$; n.s. not significant). (**B**) Representative immunoblot images of HEK 293T cells transfected with SARS2-E fused with YFP (2E-YFP). Anti-SARS2-E (2E-N, clone, N2A5E8, left), GFP (right) and GAPDH antibodies (as loading control, bottom) were used. The rat monoclonal antibody (2E-N mAb, clone N2A5E8) was produced using MY18 peptide conjugated to Keyhole limpet haemocyanin (KLH) as the antigen. (**C**) ELISA assay for the comparison of binding capacity of the anti-SARS2-E monoclonal antibody to wild-type (WT) and 2ED (EE7-8DD) mutant TAT-MY18 peptides. (**D**) The stability test of iPep-SARS2-E (TAT-MY18-2ED) using the same ELISA assay with anti-SARS2-E antibody. The peptide was incubated at 37°C in phosphate buffered solution (PBS). (**E-G**) The toxicity test of iPep-SARS2-E (TAT-MY18-2ED, 10μM, 48hr) using Jurkat cells and flowcytometry with apoptosis/necrosis assay. Healthy cells (**E**, %), apoptotic cells (**F**) and dead/necrotic cells (**G**) were counted. Camptothecin (10μM, 3hr) was used as a positive control. One-way ANOVA with Dunnett's multiple comparisons test was used (**** $P < 0.0001$; n.s. not significant, compared to non-treated). (**H**) Immunoprecipitation of 2E protein using Ni column and HEK 293T cells transfected using 2E-YFP with 6xHis-MY18-2ED (2ED) or 6xHis-MY18 wild-type constructs (WT). Anti-GFP antibody was used to blot 2E-YFP protein bands. The data underlying this figure can be found in S1 Data. All the graphs in the figure, except S2C Fig, are mean ± s.d. S2C Fig uses single-sample datasets.
