# Supplementary material for: Developing inhibitory peptides against SARS-CoV-2 envelope protein
Source: PLoS Biol. 2024 Mar 14;22(3):e3002522. doi: 10.1371/journal.pbio.3002522 (PMC10939250; doi:10.1371/journal.pbio.3002522)
Supplement: S3 Fig — (A) Representative GAPDH immunoblot image of HEK 293T cells transfected with SARS2-E fused with YFP (2E-YFP) and treated with 10 μM TAT-MY18-2ED or MY18-WT (negative control). The image between 30 and 40 kDa is used in Fig 3E. (B) Representative immunoblot images of HEK 293T cells transfected with YFP plasmid and treated with 10 μM TAT-MY18-2ED or MY18-WT (negative control) for 48 h. Anti-GFP (for YFP, top) and GAPDH antibodies (as loading control, bottom) were used. The short- and long-exposure film images are shown. #, nonspecific bands around 40 and 50 kDa are found in the cell lysate. The band images are used in Fig 3H. (C) Quantification of YFP protein expression of HEK 293T cells transfected using YFP plasmid non-treated (n = 3) and treated with TAT-MY18-2ED (n = 3) or MY18-WT peptides (n = 3). One-way ANOVA with Tukey’s multiple comparisons test was used (n.s., not significant). The data underlying this figure can be found in S1 Data. The graph in the figure is mean ± SD. (PDF) [file pbio.3002522.s003.pdf]

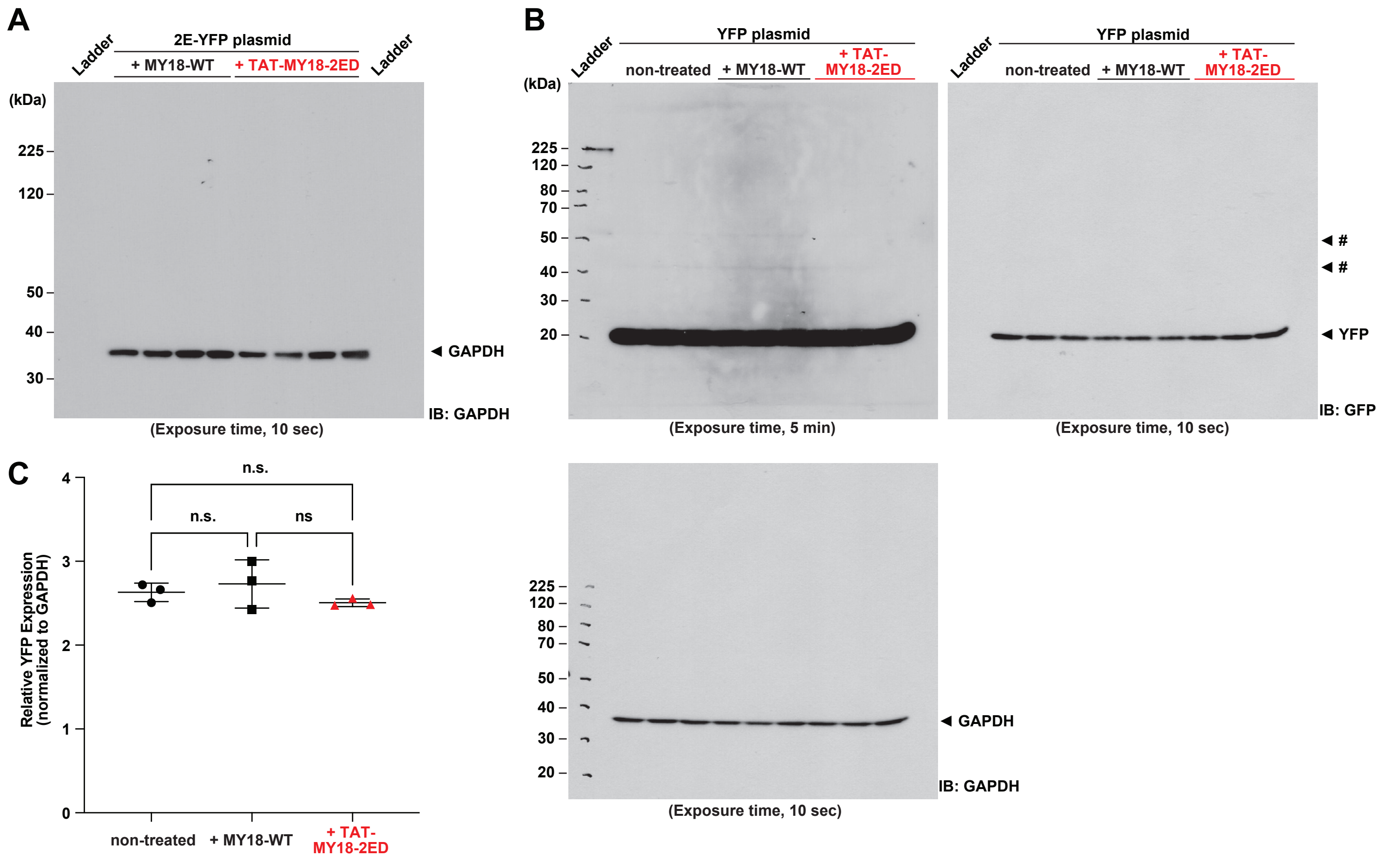

S3 Fig | The effect of iPep-SARS2-E on SARS2-E expression. (**A**) Representative GAPDH immunoblot image of HEK 293T cells transfected with SARS2-E fused with YFP (2E-YFP) and treated with 10μM TAT-MY18-2ED or MY18-WT (negative control) for 48 hours. The image between 30 and 40kDa is used in Fig 3E. (**B**) Representative immunoblot images of HEK 293T cells transfected with YFP plasmid and treated with 10μM TAT-MY18-2ED or MY18-WT (negative control). Anti-GFP (for YFP, top) and GAPDH antibodies (as loading control, bottom) were used. The short- and long-exposure film images are shown. #, non-specific bands around 40 and 50kDa are found in the cell lysate. The band images are used in Fig 3H. (**C**) Quantification of YFP protein expression of HEK 293T cells transfected using YFP plasmid non-treated (*n*=3) and treated with TAT-MY18-2ED (*n*=3) or MY18-WT peptides (*n*=3). One-way ANOVA with Tukey's multiple comparisons test was used (n.s., not significant). The data underlying this figure can be found in S1 Data. The graph in the figure is mean ± s.d.,
