# Supplementary material for: Developing inhibitory peptides against SARS-CoV-2 envelope protein
Source: PLoS Biol. 2024 Mar 14;22(3):e3002522. doi: 10.1371/journal.pbio.3002522 (PMC10939250; doi:10.1371/journal.pbio.3002522)
Supplement: S4 Fig — (A) Representative fluorescent and bright field images of time-course cell-penetrating test using Alexa Fluor 594(A594)-conjugated iPep-SARS2-E peptides, A594-TAT-MY18-2ED (amino-terminal conjugation, N-term, 10 μM, bottom), and TAT-MY18-2ED-A594 (carboxyl-terminal, C-term, 10 μM, top) in NIH 3T3 cells after the incubation started. Scale bar, 50 μm. (B) Quantification of red fluorescence-positive cells treated with the A594-conjugated peptides for the peptide cell-penetrating “on” kinetics (mean ± SD). The data underlying this figure can be found in S1 Data. (C) Experimental design for the peptide stability, “off” kinetics, quantification. (D) Representative fluorescent and bright field images after washout of A594-conjugated TAT-MY18-2ED peptide (C-term version) in NIH 3T3 cells. White arrowheads, fluorescent puncta. Scale bar, 50 μm. (PDF) [file pbio.3002522.s004.pdf]

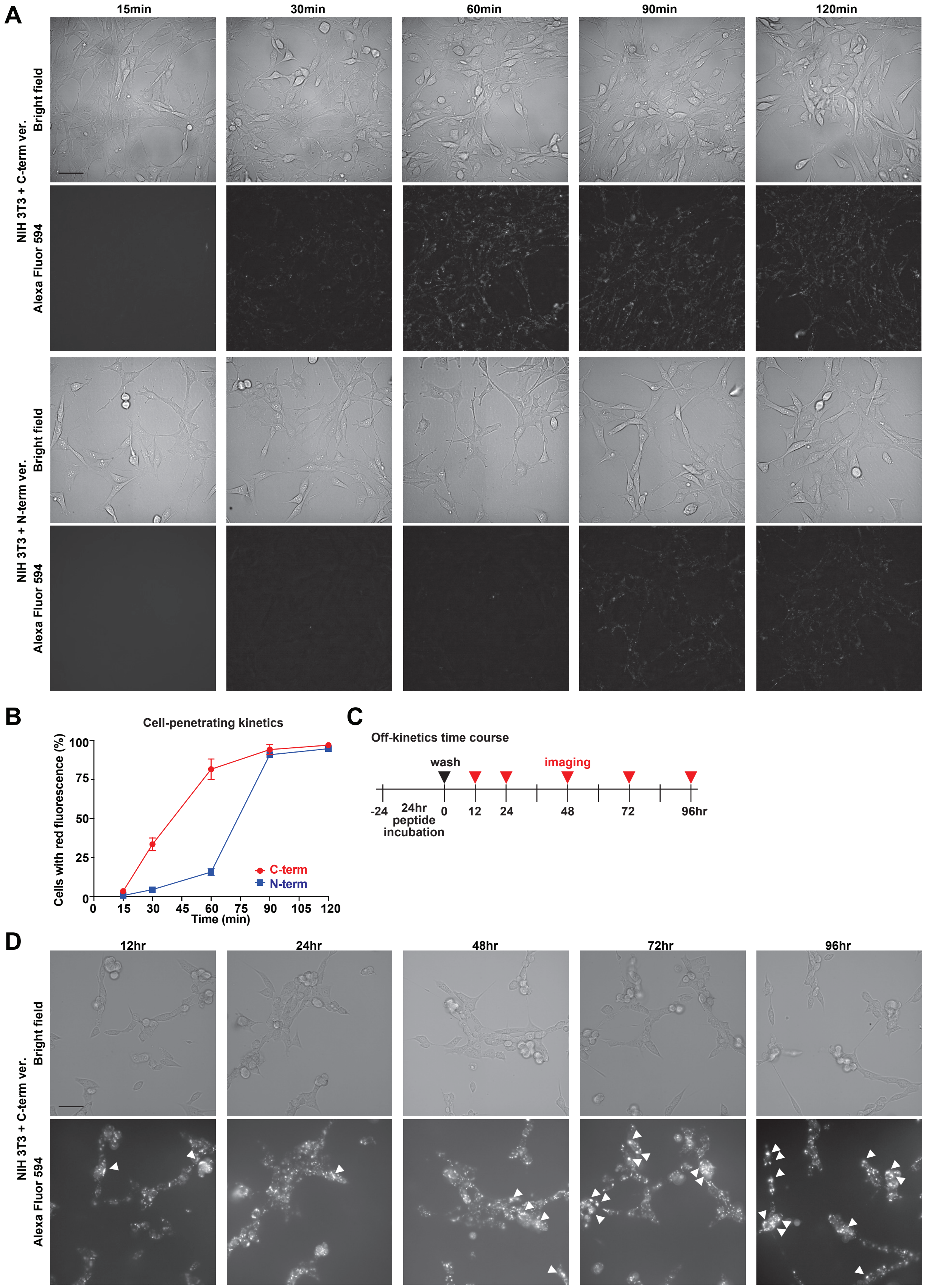

S4 Fig | Permeability of iPep-SARS2-E. (**A**) Representative fluorescent and bright field images of time-course cell-penetrating test using Alexa Fluor 594(A594)-conjugated iPep-SARS2-E peptides, A594-TAT-MY18-2ED (amino-terminal conjugation, N-term, 10μM, bottom) and TAT-MY18-2ED-A594 (carboxyl-terminal, C-term, 10μM, top) in NIH 3T3 cells after the incubation started. Scale bar, 50μm. (**B**) Quantification of red fluorescence-positive cells treated with the A594-conjugated peptides for the peptide cell-penetrating "on" kinetics (mean ± s.d.). The data underlying this figure can be found in S1 Data. (**C**) Experimental design for the peptide stability, "off" kinetics, quantification. (**D**) Representative fluorescent and bright field images after washout of A594-conjugated TAT-MY18-2ED peptide (C-term version) in NIH 3T3 cells. White arrowheads, fluorescent puncta. Scale bar, 50μm.
