# Supplementary material for: Developing inhibitory peptides against SARS-CoV-2 envelope protein
Source: PLoS Biol. 2024 Mar 14;22(3):e3002522. doi: 10.1371/journal.pbio.3002522 (PMC10939250; doi:10.1371/journal.pbio.3002522)
Supplement: S5 Fig — (A) Representative red fluorescent and bright field images of Vero-E6 cells incubated with TAT-MY18-2ED-AlexaFluor594 (C-term conjugated version, 10 μM). Scale bars, 50 μm. (B) Quantification of red fluorescence-positive Vero-E6 cells treated with the AlexaFluor594-conjugated peptides (C-term conjugated version) for measuring the peptide cell-penetrating “on” kinetics (mean ± SD). The data underlying this figure can be found in S1 Data. (PDF) [file pbio.3002522.s005.pdf]

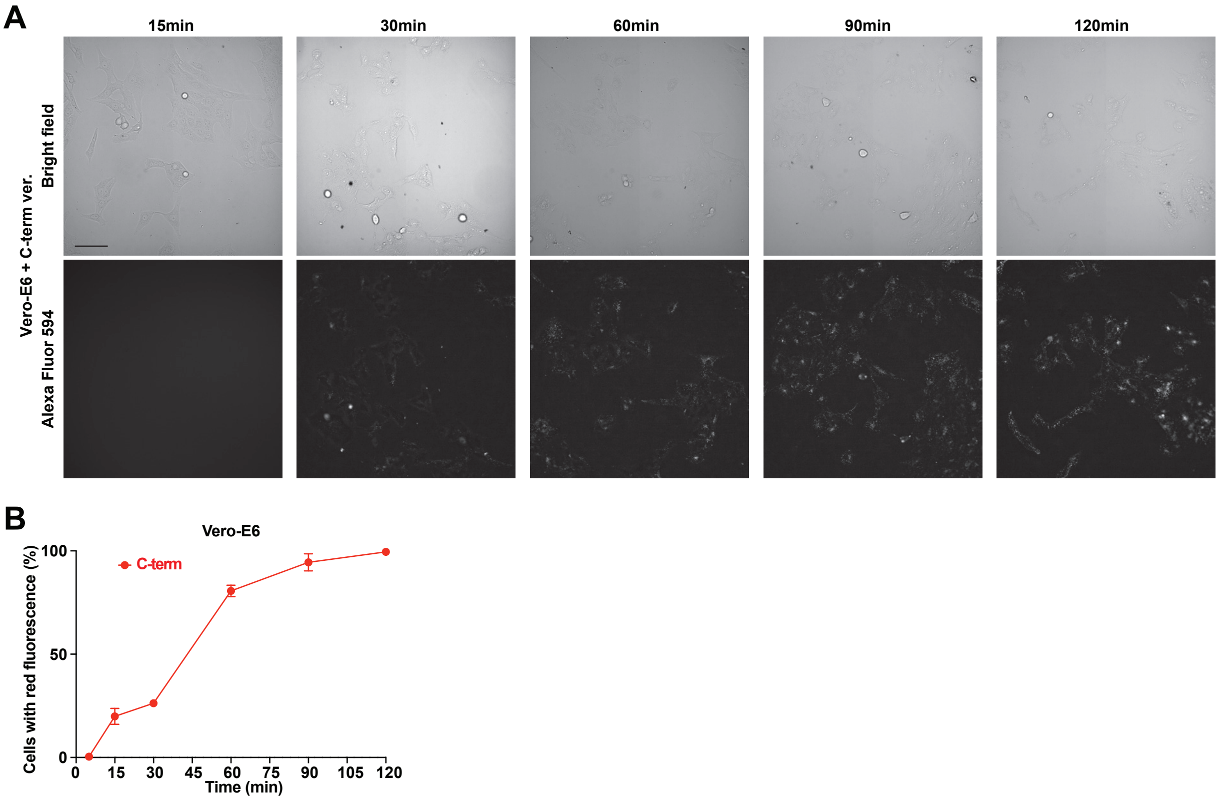

S5 Fig | Cell penetration of iPep-SARS2-E in Vero-E6 cells (**A**) Representative red fluorescent and bright field images of Vero-E6 cells incubated with TAT-MY18-2ED-AlexaFluor594 (C-term conjugated version, 10 μM). Scale bars, 50 μm. (**B**) Quantification of red fluorescence-positive Vero-E6 cells treated with the AlexaFluor594-conjugated peptides (C-term conjugated version) for measuring the peptide cell-penetrating "on" kinetics (mean ± s.d.). The data underlying this figure can be found in S1 Data.
