# Supplementary material for: Developing inhibitory peptides against SARS-CoV-2 envelope protein
Source: PLoS Biol. 2024 Mar 14;22(3):e3002522. doi: 10.1371/journal.pbio.3002522 (PMC10939250; doi:10.1371/journal.pbio.3002522)
Supplement: S6 Fig — (A) Electron microscopic image of iPep-SARS2-E-treated Vero-E6 cells at 24 h post-infection. Arrowheads, small particles found in the nuclear envelope. Scale bar, 1 μm. (B) Experimental design to examine whether intracellular particles are infectious in Vero-E6 cells treated with iPep-SARS2-E. (C) Result of quantitative endpoint titration assay used to quantify intracellular virus particles of PBS- and iPep-SARS2-E (10 μM)-treated Vero-E6 cells. There is a significant reduction of infectivity in iPep-SARS2-E though still infectious. Each well (cell plating at 24 h, 2,500 harvested cells onto 4 × 10^4^ uninfected fresh cells per a well that were seeded the night before) was scored based on infectivity compared to virus controls with zero indicating no infection and 100 indicating complete infection (CPE). Student’s t test was used (**** P < 0.0001). (D) Western blots of Spike and GAPDH proteins in PBS- and iPep-SARS2-E (10 μM)-treated Vero-E6 cells at 24 h post-infection with SARS-CoV-2 WA1 (MOI, 0.10, 24 h), suggesting the effect of iPep-SARS2-E on Spike expression and/or stability. (E–H) qPCR of SARS-CoV-2 N (E), E (F), JUN/AP-1 expression (G) of PBS (n = 6)- and iPep-SARS2-E (10 μM, n = 6)-treated Vero-E6 cells comparing to non-infected cells (n = 6) at 48 h post-infection. The expression of these genes was normalized to a house-keeping gene, GAPDH. One-way ANOVA with Tukey’s multiple comparisons test was used (**** P < 0.0001; *** P < 0.001; ** P < 0.01; n.s., not significant). (H) qPCR of SARS2-CoV-2 N expression of PBS (n = 6)- and iPep-SARS2-E (10 μM, n = 6)-treated Vero-E6 cell culture supernatant (sup) at 48 h post-infection. Student’s t test was used (**** P < 0.0001). The cDNA samples of cells and cell culture sup were prepared with TRIzol Plus RNA Purification kit, PureLink DNase set and SuperScript III and then diluted (1/5) using UltraPure distilled water for conducting qPCR. (I) Representative confocal fluorescent images of Vero-E6 cells treated with PBS [file pbio.3002522.s006.pdf]

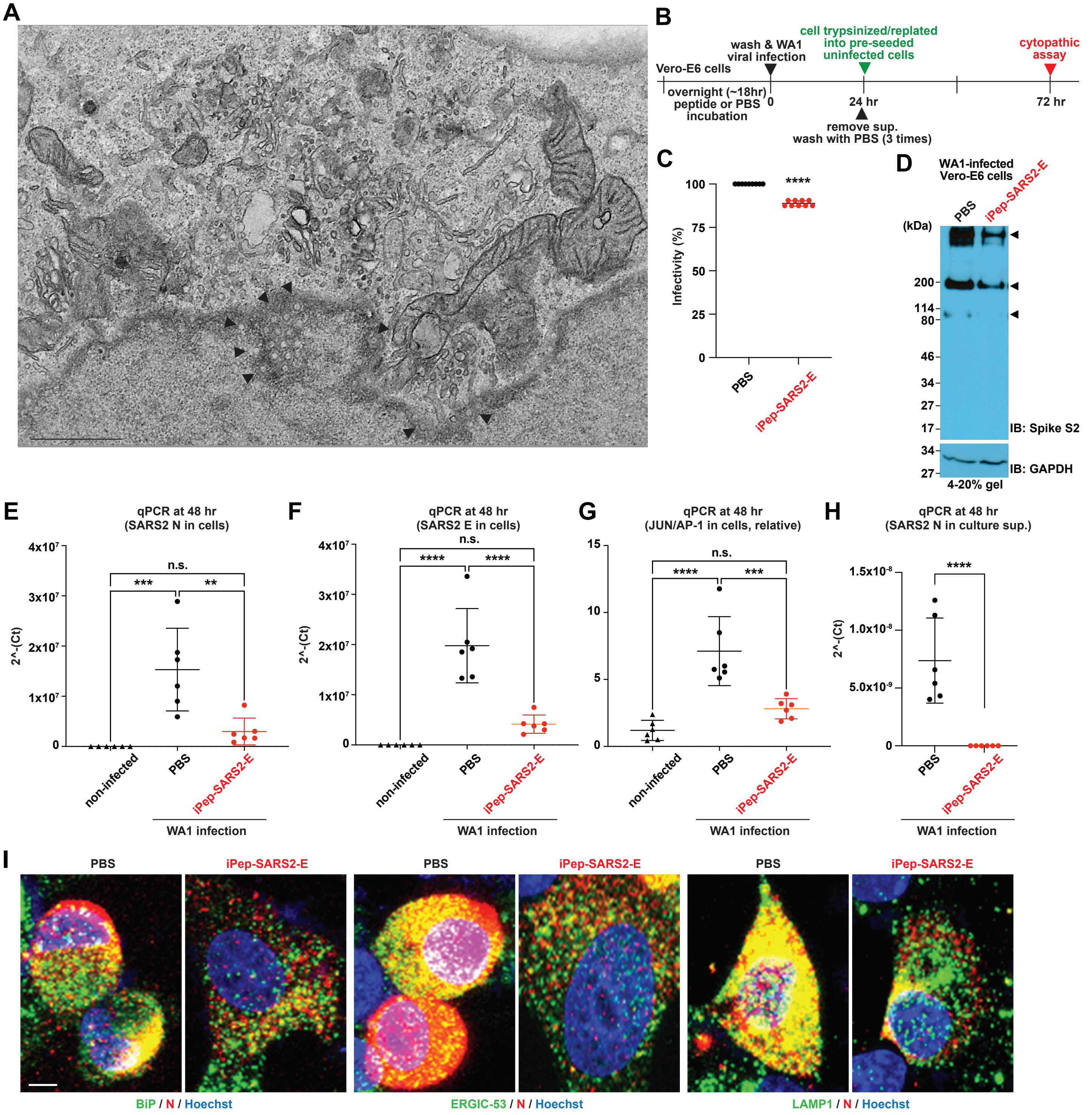

S6 Fig | iPep-SARS2-E *in vitro* validation. (**A**) Electron microscopic image of iPep-SARS2-E-treated Vero-E6 cells at 24 hours post-infection. Arrowheads, small particles found in the nuclear envelope. Scale bar, 1μm. (**B**) Experimental design to examine whether intracellular particles are infectious in Vero-E6 cells treated with iPep-SARS2-E. (**C**) Result of quantitative endpoint titration asssay used to quantify intracellular virus particles of PBS- and iPep-SARS2-E (10μM)-treated Vero-E6 cells. There is a significant reduction of infectivity in iPep-SARS2-E though still infectious. Each well (cell plating at 24hr, 2,500 harvested live cells onto $4x10^4$ uninfected fresh cells per a well that were seeded the night before) was scored based on infectivity compared to virus controls with zero indicating no infection and 100 indicating complete infection (CPE). Student's *t*-test was used (**** $P < 0.0001$). (**D**) Western blots of Spike and GAPDH proteins in PBS- and iPep-SARS2-E (10μM)-treated Vero-E6 cells at 24 hours post-infection with SARS-CoV-2 WA1 (MOI, 0.10, 24hr), suggesting the effect of iPep-SARS2-E on Spike expression and/or stability. (**E-H**) qPCR of SARS-CoV-2 N (**E**), E (**F**), JUN/AP-1 expression (**G**) of PBS (n=6)- and iPep-SARS2-E (10μM, n=6)-treated Vero-E6 cells comparing to non-infected cells (n=6) at 48hr post-infection. The expression of these genes was normalized to a house-keeping gene, GAPDH. One-way ANOVA with Tukey's multiple comparisons test was used (**** $P < 0.0001$; *** $P < 0.001$; ** $P < 0.01$; n.s., not significant). (**H**) qPCR of SARS2-CoV-2 N expression of PBS (n=6)- and iPep-SARS2-E (10μM, n=6)-treated Vero-E6 cell culture supernatant (sup) at 48hr post-infection. Student's *t*-test was used (**** $P < 0.0001$). The cDNA samples of cells and cell culture sup were prepared with TRIzol Plus RNA Purification kit, PureLink DNase set and SuperScript III and then diluted (1/5) using UltraPure distilled water for conducting qPCR. (**I**) Representative confocal fluorescent images of Vero-E6 cells treated with PBS or iPep-SARS2-E at 48hr post-infection. SARS-CoV-2 N antibody (red) and Hoechst 33258 dye (blue, for nucleus) were used with antibodies of subcellular organelle markers (green): BiP for endoplasmic reticulum (ER), ERGIC-53 for ER Golgi inter compartment (ERGIC) and LAMP1 for lysosome. Scale bar, 5μm. The data underlying this figure can be found in S1 Data. All the graphs in the figure are mean ± s.d.
