# Supplementary material for: Developing inhibitory peptides against SARS-CoV-2 envelope protein
Source: PLoS Biol. 2024 Mar 14;22(3):e3002522. doi: 10.1371/journal.pbio.3002522 (PMC10939250; doi:10.1371/journal.pbio.3002522)
Supplement: S7 Fig — (A) Schematic representative of mouse intranasal administration of iPep-SARS2-E. Red box demonstrates the nasal tissue region harvested for the following fluorescent imaging. The image is from BioRender software. (B) Representative fluorescent and bright field images of nasal tissues isolated from mice administrated intranasally with PBS or Alexa594-conjugated iPep-SARS2-E peptide (TAT-MY18-2ED-A594, 10 μM, 2 h). After isolating the tissues, the samples were washing using PBS 3 times, and the fluorescent and bright field images were taken by a fluorescent stereoscope. Scale bar, 1 mm. (C) Experimental design for the iPep-SARS2-E safety test in vivo. (D) There is no significant difference the effects on body weight among iPep-SARS2-E-treated (n = 6), non-treated (n = 5), and PBS-treated Balb/c mouse groups (n = 5). One-way ANOVA with Tukey’s multiple comparisons was used at each day. (E, F) There were no significant differences in Cxcl12 (E) and C5a (F) among iPep-SARS2-E-treated (n = 5), non-treated (n = 4), and PBS-treated mice (n = 4). One-way ANOVA with Tukey’s multiple comparisons was used (n.s., not significant). (G) Experimental design for the iPep-SARS2-E test in vivo using intranasal administration. (H) iPep-SARS2-E prevents body weight loss in SARS-CoV-2 MA10-infected Balb/c mice (5.0 × 10^4 PFU/mouse). Student’s t test was used at each day (** P < 0.01; * P < 0.05). (I, J) Representative immunoblots of SARS-CoV-2 E (2E, I) and mouse Gapdh proteins (J) in SARS-CoV-2 MA10-infected mouse lung tissues with PBS or iPep-SARS2-E treatment. (K) iPep-SARS2-E peptides significantly reduced the protein expression of 2E in MA10-infected Balb/c mouse lung tissues (PBS, n = 4; iPep-SARS2-E, n = 4). Student’s t test was used (* P < 0.05). The data underlying this figure can be found in S1 Data. All the graphs in the figure are mean ± SD. (PDF) [file pbio.3002522.s007.pdf]

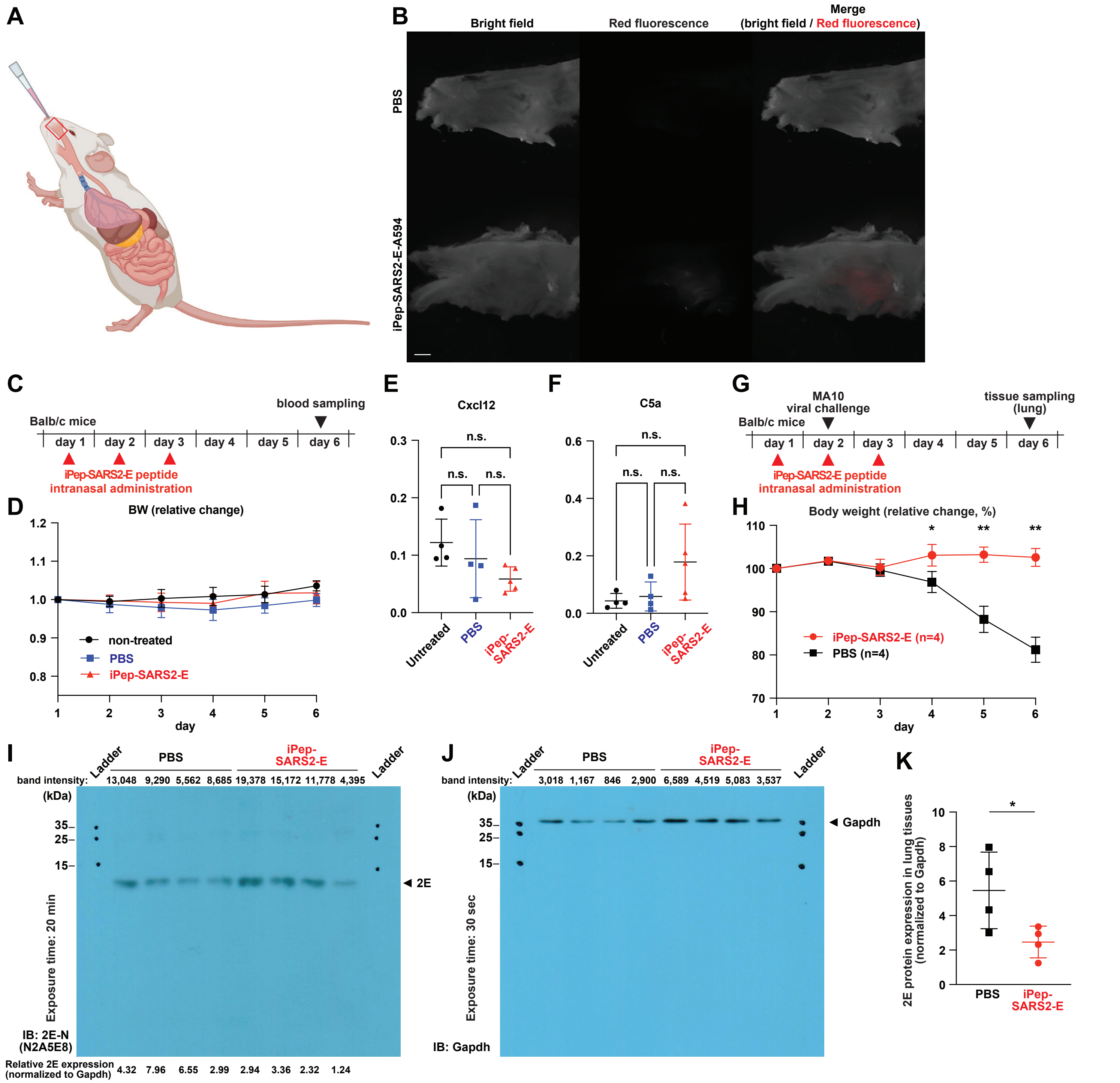

S7 Fig | iPep-SARS2-E *in vivo* test using intranasal administration. (**A**) Schematic representative of mouse intranasal administration of iPep-SARS2-E. Red box demonstrates the nasal tissue region harvested for the following fluorescent imaging. The image is from BioRender software. (**B**) Representative fluorescent and bright field images of nasal tissues isolated from mice administrated intranasally with PBS or Alexa594-conjugated iPep-SARS2-E peptide (TAT-MY18-2ED-A594, 10 μM, 2hr). After isolating the tissues, the samples were washing using PBS three times, and the fluorescent and bright field images were taken by a fluorescent stereoscope. Scale bar, 1mm. (**C**) Experimental design for the iPep-SARS2-E safety test *in vivo*. (**D**) There is no significant difference the effects on body weight among iPep-SARS2-E-treated (n=6), non-treated (n=5) and PBS-treated Balb/c mouse groups (n=5). One-way ANOVA with Tukey's multiple comparisons was used at each day. (**E-F**) There were no significant differences in Cxcl12 (**E**) and C5a (**F**) among iPep-SARS2-E-treated (n=5), non-treated (n=4) and PBS-treated mice (n=4). One-way ANOVA with Tukey's multiple comparisons was used (n.s. not significant). (**G**) Experimental design for the iPep-SARS2-E test *in vivo* using intranasal administration. (**H**) iPep-SARS2-E prevents body weight loss in SARS-CoV-2 MA10-infected Balb/c mice (5.0x10^4 PFU/mouse). Student's t-test was used at each day (** $P < 0.01$; * $P < 0.05$). (**I-J**) Representative immunoblots of SARS-CoV-2 E (2E, **I**) and Gapdh proteins (**J**) in SARS-CoV-2 MA10-infected mouse lung tissues with PBS or iPep-SARS2-E treatment. (**K**) iPep-SARS2-E significantly reduced the protein expression of 2E in MA10-infected Balb/c mouse lung tissues (PBS, n=4; iPep-SARS2-E, n=4). Student's t-test was used (* $P < 0.05$). The data underlying this figure can be found in S1 Data. All the graphs in the figure are mean ± s.d.
