# Supplementary material for: Developing inhibitory peptides against SARS-CoV-2 envelope protein
Source: PLoS Biol. 2024 Mar 14;22(3):e3002522. doi: 10.1371/journal.pbio.3002522 (PMC10939250; doi:10.1371/journal.pbio.3002522)
Supplement: S8 Fig — (A) Alignment of MY18 WT and mutant candidate sequences (MT, targeted amino acids, underline), following the result using the MY18 deletion constructs (Fig 2F) and mutagenesis. (B) Testing iPep-SARS2-E negative control (neg. Ctrl) mutant constructs using NFAT/AP-1 assay in mock- or 2E-transfected HEK 293T cells. One-way ANOVA with Tukey’s multiple comparisons test (**** P < 0.0001; * P < 0.05; n.s., not significant). (C) Relative fluorescent intensity of DND-189 dye in NIH 3T3 cells transfected using mock or 2E-mKate2 plasmid without (-) and with the neg. Ctrl peptide constructs. One-way ANOVA with Tukey’s multiple comparisons test (**** P < 0.0001; *** P < 0.001; n.s., not significant). (D) qPCR of SARS2 N expression of the neg. Ctrl mutant peptide-, iPep-SARS2-E-, and PBS- treated Vero E6 cell culture supernatant. All the peptides (10 μM) were used overnight (approximately 18 h) and then washed before SARS-CoV-2 WA1 infection. One-way ANOVA with Tukey’s multiple comparisons test (** P < 0.01; n.s., not significant). (E) Representative images of phase contrast and yellow fluorescence of Vero-E6 cells infected with pseudo virus (MOI, 0.05) produced by SARS2 Spike, E, M, dR8.2 and YFP reporter using iPep-SARS2-E treatment (10 μM). The neg. Ctrl mutant peptide (10 μM) was used as a negative control. Scale bar, 20 μm. (F) There is no significant difference in YFP-positive cells between iPep-SARS2-E and neg. Ctrl, suggesting no effect of iPep-SARS2-E on the virus entry. Student’s t test was used (n.s., not significant). (G) Experimental design of the iPep-SARS2-E intranasal administration with the neg. Ctrl mutant peptide as a negative control in vivo. (H) Body weight changes of the mouse groups. Student’s t test was used (* P < 0.05). (I) There is a significant reduction of lung viral titer in iPep-SARS2-E-treated mice compared to the neg. Ctrl. Median tissue culture infection dose (TCID) is normalized to lung wet weight (g) measured before the tissue homogenization to [file pbio.3002522.s008.pdf]

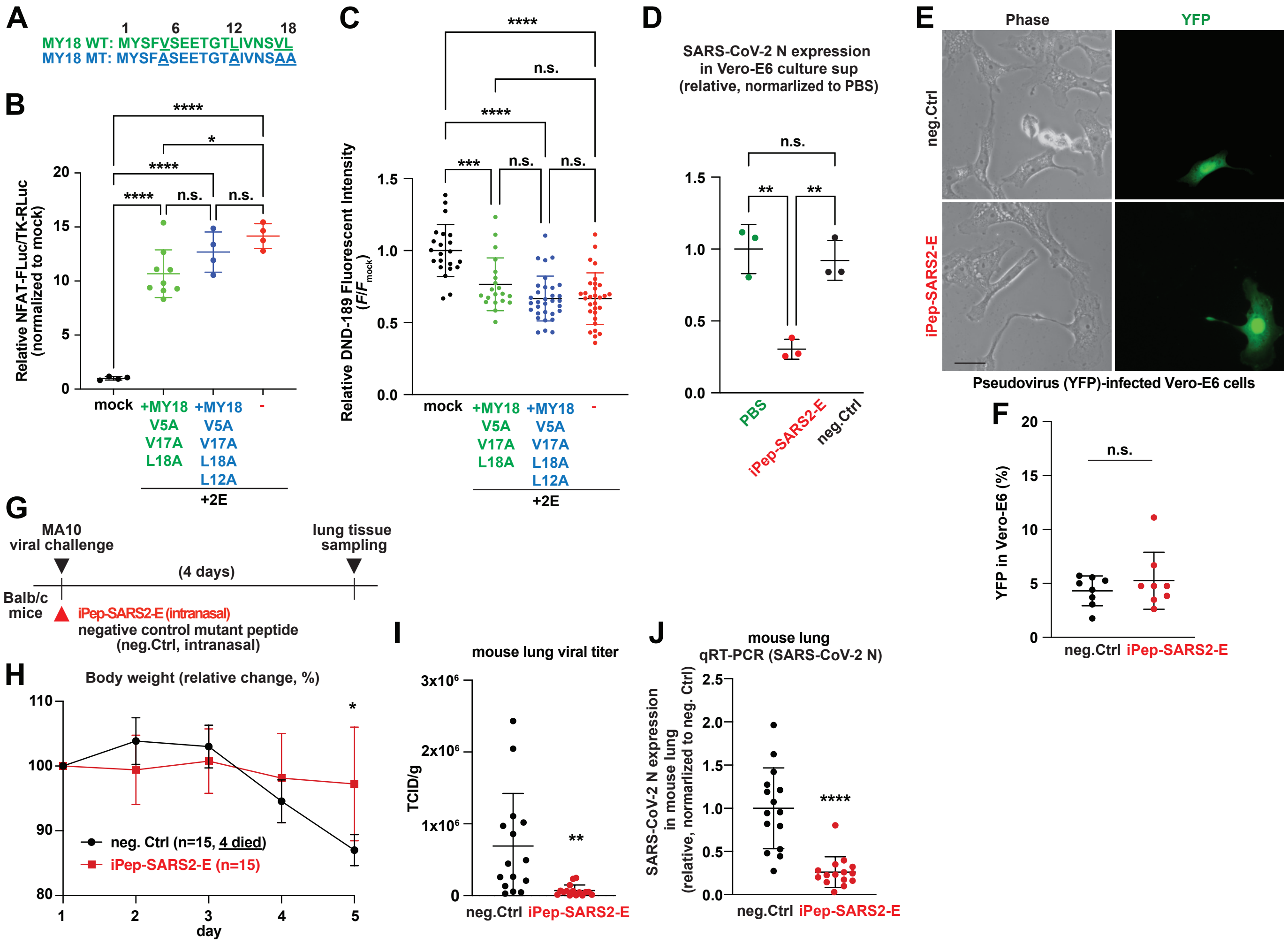

S8 Fig | iPep-SARS2-E and negative control peptide test i*n vitro* and *in vivo*. (**A**) Alignment of MY18 WT and mutant candidate sequences (MT, targeted amino acids, underline), following the result using the MY18 deletion constructs (Fig 2F) and mutagenesis. (**B**) Testing iPep-SARS2-E negative control (neg. Ctrl) mutant constructs using NFAT/AP-1 assay in mock- or 2E-transfected HEK 293T cells. One-way ANOVA with Tukey's multiple comparisons test (**** $P < 0.0001$; * $P < 0.05$; n.s. not significant). (**C**) Relative fluorescent intensity of DND-189 dye in NIH 3T3 cells transfected using mock or 2E-mKate2 plasmid without (-) and with the neg. Ctrl peptide constructs. One-way ANOVA with Tukey's multiple comparisons test (**** $P < 0.0001$; *** $P < 0.001$; n.s. not significant). (**D**) qPCR of SARS2 N expression of the neg. Ctrl mutant peptide-, iPep-SARS2-E- and PBS- treated Vero E6 cell culture supernatant. All the peptides (10μM) were used overnight (~18hr) and then washed before SARS-CoV-2 WA1 infection. One-way ANOVA with Tukey's multiple comparisons test (** $P < 0.01$; n.s. not significant). (**E**) Representative images of phase contrast and yellow fluorescence of Vero-E6 cells infected with pseudo virus (MOI, 0.05) produced by SARS2 Spike, E, M, dR8.2 and YFP reporter using iPep-SARS2-E treatment (10μM). The neg. Ctrl mutant peptide (10μM) was used as a negative control. Scale bar, 20μm. (**F**) There is no significant difference in YFP-positive cells between iPep-SARS2-E and neg. Ctrl, suggesting no effect of iPep-SARS2-E on the virus entry. Student's *t*-test was used (n.s. not significant). (**G**) Experimental design of the iPep-SARS2-E intranasal administration with the neg. Ctrl mutant peptide as a negative control *in vivo*. (**H**) Body weight changes of the mouse groups. Student's *t*-test was used (* $P < 0.05$). (**I**) There is a significant reduction of lung viral titer in iPep-SARS2-E-treated mice compared to the neg. Ctrl. Median tissue culture infection dose (TCID) is normalized to lung wet weight (g) measured before the tissue homogenization to isolate the virus. (**J**) iPep-SARS2-E significantly reduced the transcript expression of SARS-CoV-2 N in MA10-infected Balb/c mouse lung tissues. Student's *t*-test was used (**** $P < 0.0001$; ** $P < 0.01$). The data underlying this figure can be found in S1 Data. All the graphs in the figure are mean ± s.d.
