# Supplementary material for: Developing inhibitory peptides against SARS-CoV-2 envelope protein
Source: PLoS Biol. 2024 Mar 14;22(3):e3002522. doi: 10.1371/journal.pbio.3002522 (PMC10939250; doi:10.1371/journal.pbio.3002522)
Supplement: S9 Fig — Envelope protein sequence alignment of SARS-CoV-2, MERS-CoV, HCoV-229E, HCoV-NL63, HCoV-OC43, and HCoV-HKU1. CLUSTALW 2.1 multiple sequence alignment software is used to obtain the alignment. (PDF) [file pbio.3002522.s009.pdf]

```
SARS-CoV-2      --MYSFVSEETGTLIVNSVLLFLAFVVFLLVTLAILTALRLCAYCCNIVNVSLVKPSFYV
MERS-CoV        --MLPFVQERIGLFIVNFFIFTVVCAITLLVCMAFLTATRLCVQCMTGFNTLLVQPALYL
HCoV-OC43       MFMADAYLADTVWYVGQIIFIVAICLLVTIVVVAFLATFKLCIQLCGMCNTLVLSPSIYV
HCoV-HKU1       --MVDLFFNDTAWYIGQILVLVLFCLISLIFVVAFLATIKLCMQLCGFCNFFIISPSAYV
HCoV-229E       --MFLKLVDDHA-LVVNVLLWCVVLIVILLVCITIIKLIKLCFTCHMFCNRTVYGPIKNV
HCoV-NL63       --MFLRLIDDNG-IVLNSILWLLVMIFFFVLAMTFIKLIQLCFTCHYFFSRTLYQPVYKI
                              : : ..      .  :. ::::   :**       .  :  *   :

SARS-CoV-2      YSR-----VKNLNSSR--VPDLLV
MERS-CoV        YNTGRSVYVKFQDSKPPLPPDEWV
HCoV-OC43       FNRGRQFYEFYNDVKPPVLDVDDV
HCoV-HKU1       YKRGMQLYKSYSEQVIPPTSDYLI
HCoV-229E       YHI----YQSYMHIDPFPKRVIDF
HCoV-NL63       FLA----YQDYMQIAPVPAEVLNV
                :           .          .
```

S9 Fig | Alignment of human coronavirus envelope proteins. Envelope protein sequence alignment of SARS-CoV-2, MERS-CoV, HCoV-229E, HCoV-NL63, HCoV-OC43 and HCoV-HKU1. CLUSTALW 2.1 multiple sequence alignment software is used to obtain the alignment.
