# Supplementary material for: Developing inhibitory peptides against SARS-CoV-2 envelope protein
Source: PLoS Biol. 2024 Mar 14;22(3):e3002522. doi: 10.1371/journal.pbio.3002522 (PMC10939250; doi:10.1371/journal.pbio.3002522)
Supplement: S1 Raw Images — (PDF) [file pbio.3002522.s011.pdf]

Bekdash *et al.* 2024 *PLOS Biol.*

Notes of the original blotting images

Fig 3A: see the below (page 2)

Fig 3E top: the whole PVDF image was used for the 2E-YFP blot.

Fig 3E bottom: the whole PVDF image is shown for the GAPDH blot in S3A Fig.

Fig 3H bottom: the whole PVDF images are shown for the YFP and GAPDH blots in S3B Fig.

S2B Fig: see the below (page 3)

S2H Fig: see the below (page 4)

S6D Fig: see the below (page 5)

S7I Fig: the whole PVDF image was used for the 2E blot.

S7J Fig: the whole PVDF image was used for the Gapdh blot.

Fig 3A

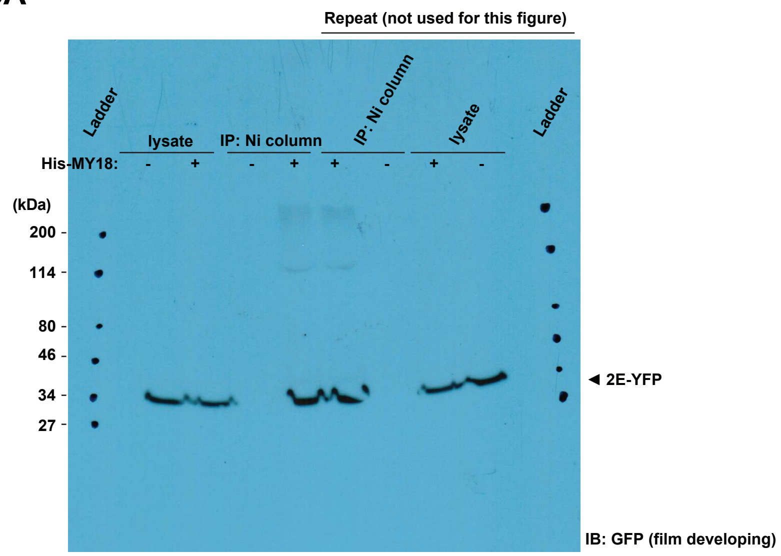
Repeat (not used for this figure)
Ladder
lysate
IP: Ni column
IP: Ni column
lysate
Ladder
His-MY18:
-
+
-
+
+
-
+
-
(kDa)
200
114
80
46
34
27
◄ 2E-YFP
IB: GFP (film developing)

S2B Fig

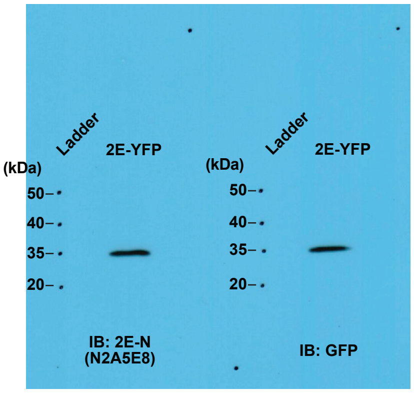

(The PVDFs were cut for each blotting; film developing)

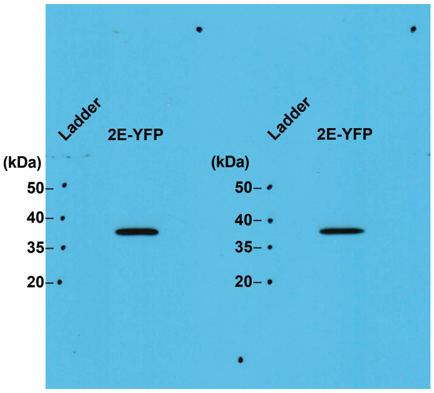

IB: GAPDH

(the above/same PVDFs were used after stripping)

S2H Fig

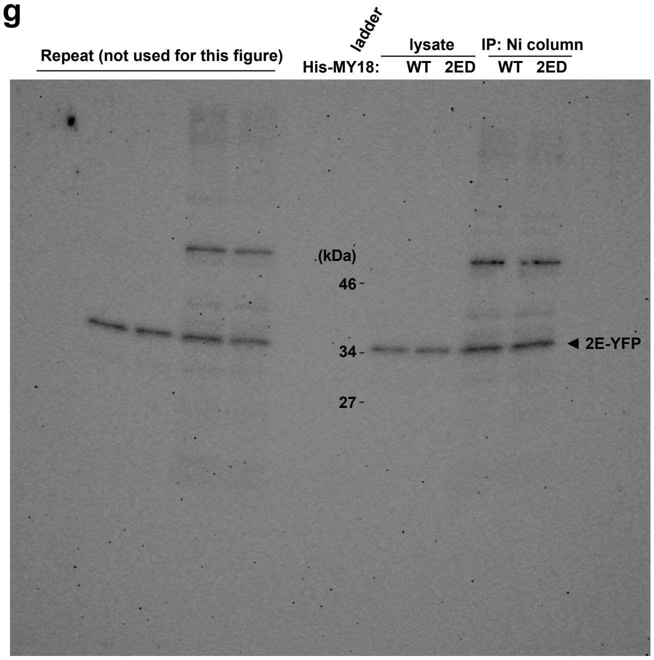

IB: GFP (chemiluminescence was detected by Bio-Rad ChemiDoc MP system)

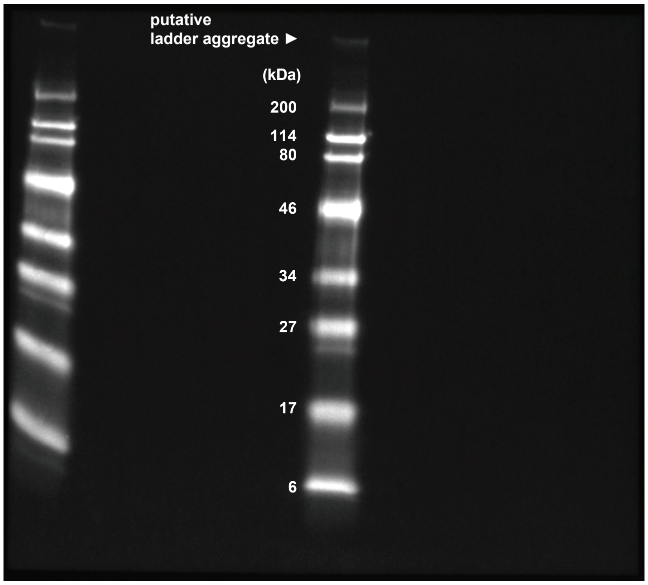

Coomassei Blue (for the ladder, the same/above PVDF was used)

S6D Fig

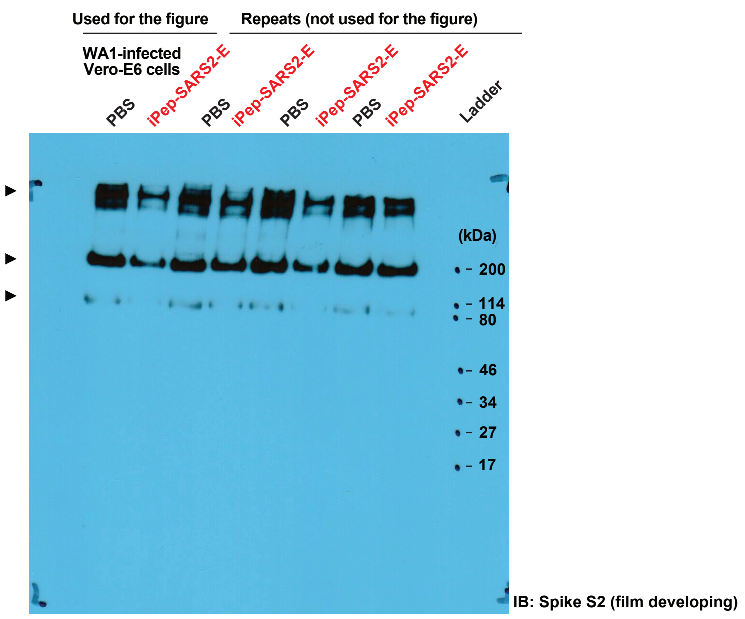
Used for the figure
Repeats (not used for the figure)
WA1-infected Vero-E6 cells
PBS
iPep-SARS2-E
PBS
iPep-SARS2-E
PBS
iPep-SARS2-E
PBS
iPep-SARS2-E
Ladder
(kDa)
200
114
80
46
34
27
17
IB: Spike S2 (film developing)

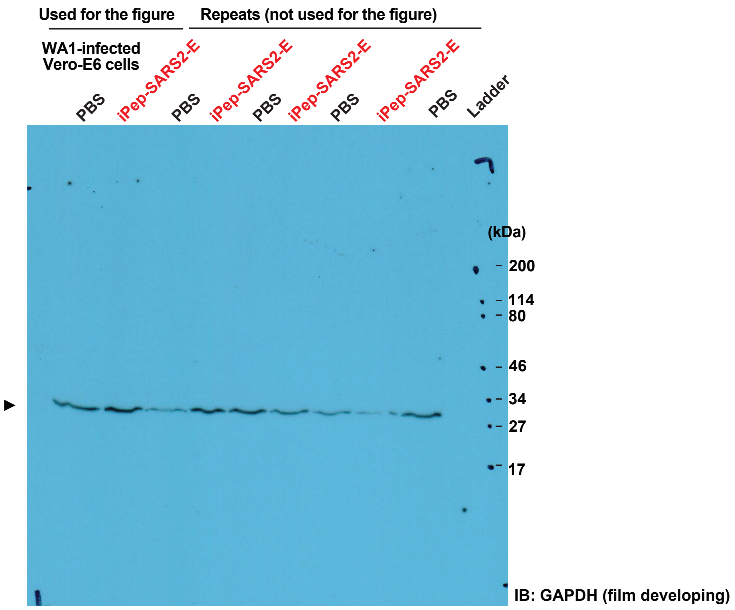
Used for the figure
Repeats (not used for the figure)
WA1-infected Vero-E6 cells
PBS
iPep-SARS2-E
PBS
iPep-SARS2-E
PBS
iPep-SARS2-E
PBS
iPep-SARS2-E
PBS
Ladder
(kDa)
200
114
80
46
34
27
17
IB: GAPDH (film developing)
