# Supplementary material for: Developing inhibitory peptides against SARS-CoV-2 envelope protein
Source: PLoS Biol. 2024 Mar 14;22(3):e3002522. doi: 10.1371/journal.pbio.3002522 (PMC10939250; doi:10.1371/journal.pbio.3002522)
Supplement: Attachment — Submitted filename: Bekdash_PLOS_Biol_2023_rebuttal_R1_final.pdf [file pbio.3002522.s012.pdf]

Reviewer #1 (Remarks to the Author):

The authors have developed a peptide based on SARS2 E protein that may have a therapeutic effect against COVID-19. This peptide (named iPepSARS2E) is based on the N-terminal 18 residues of E, and seems to inhibit lysosome acidification, whether through proton channel activity or membrane disruption, and also viral/host protein expression which is modified by E overexpression. The first is rationalized by a direct interaction of the peptide with E, and inhibition of its channel activity. The second, seems to be explained at least in part by increased degradation. Negative controls in this new version are appropriate and some evidence is provided of physical interaction of E and the short peptide.

The authors show data both in vitro and in vivo that look intriguing. Overall, the paper shows that the N-terminal domain of the E protein in SARS-2 CoV reverses changes in lysosome fluorescence and host/viral protein expression caused by E overexpression or viral infection. I find the results showing the changes caused by the peptide convincing, although the mechanism by which these are happening are not entirely clear.

We appreciate the reviewer's positive comments on the beneficial effect of iPep-SARS2-E.

It appears that the DD mutant has a direct interaction with E protein, but how is this linked to the events observed is more challenging to explain. The readouts used are sequence dependent: a double D mutant has a better effect than the WT, whereas another mutant chosen shows almost no effect.

The DD mutant was found to have higher activity, and is used in subsequent experiments. If the hypothesis of interaction is correct, this mutant should have higher affinity for E than the WT sequence of the peptide, and even more for the negative control used. Can the authors show some experimental data that this is the case? By SPR/BLI for example? The authors use electrophysiological recordings, but I am unable to comment if the current observed is due to E. The experiment may be far more convincing with a negative control E mutant which has no channel activity (N15A: refs PMID: 24788150, PMID: 22832120).

We thank this reviewer for these comments on the molecular mechanism underlying the effect of iPep-SARS2-E (TAT-MY18-2ED). Regarding the electrophysiological recording, we have shared our plasmid DNA with various electrophysiologists, and we confirmed the reproducibility in other laboratories using mammalian cell lines (unpublished) and oocyte expression system (Harrison *et al. Commun. Biol.* 2022, doi: 10.1038/s42003-022-03669-2.). In a separate manuscript, we plan to report the effect of a variety of mutants on the electrophysiological properties of Envelope proteins.

To address the hypothesis that the DD mutant (E7D & E8D, we called 2ED mutant) may have higher affinity for **SARS-CoV-2 E (2E) protein** than the wild-type, we conducted computational modeling of the full-length 2E pentameric structure with iPep-SARS2-E using AlphaFold2 ColabFold model (DeepMind). However, plDDT value of the provided structure and interaction was so low (<50, not shown) that we could not conclude and finalize their interaction interface and difference between the wild-type and 2ED mutant peptides.

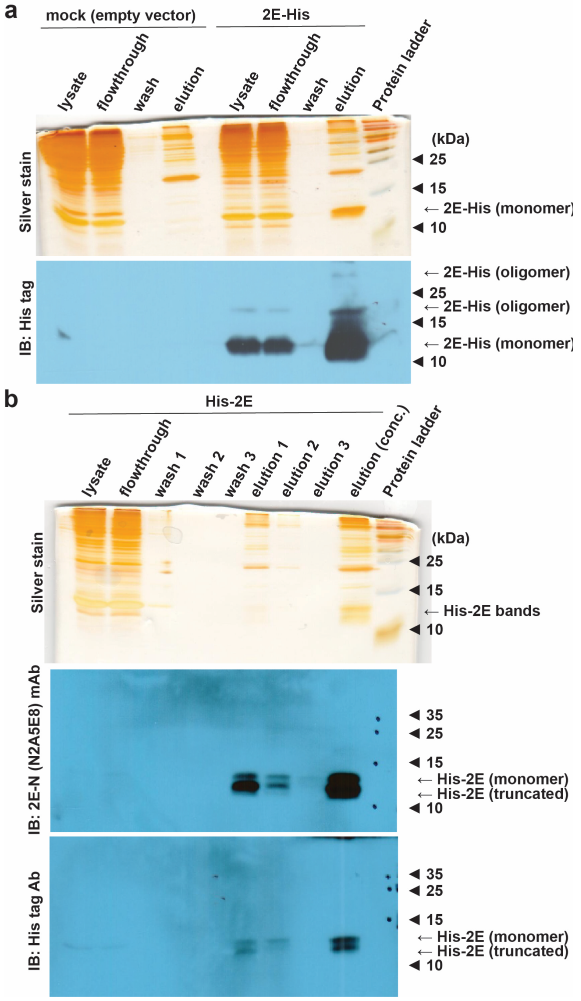

Fig. R1 | Recombinant Envelope proteins using bacterial expression system and Hig-tag/Ni column purification. Representative sliver stain and immunoblot images of SARS-CoV-2 Envelope (2E) protein using His tag: 2E-His (**a**) and His-2E constructs (**b**). Though 2E protein is composed of 75 amino acids, the band was localized between 10kDa and 15kDa protein ladders; SDS sample buffer contains 4M urea.

For setting up binding assays, next we expressed and tried to purify bacterial recombinant 2E protein:

1) His-Envelope and Envelope-His protein constructs:

We used pCold vector (TaKaRa Bio/Clontech) to express His-tagged full-length 2E proteins in Rosetta 2(DE3) bacteria strain (two versions, N- and C-terminal His-tagged). Unfortunately, the protein expression was low in bacteria and the yield was low, requiring silver gel staining, and the majority of 2E proteins were found in the flowthrough fraction, suggesting that His-tag addition to the ends might not be suitable to purify the full-length 2E proteins. Also, we found that the N- and C-terminus of His-2E proteins might be degraded/ truncated (**Fig. R1a,b, previous page**).

2) Thrombin cleavable construct:

Following the results using 2E-His and His-2E constructs, next we used the same pCold vector, thrombin cleavage site and super-folder CFP (sfCFP)-His to improve the expression, binding to Ni column and yield and to purify the full-length 2E protein using Ni column and thrombin. sfCFP was thought to be helpful when fluorescent chromatography is used, according to our previous experiences. The 2E fusion protein expression became higher in bacteria, and the yield was improved, allowing us to observe the protein bands using CBB staining (**Fig. R2a**). However, the majority of 2E fusion proteins were still found in the flowthrough fractions, and 2E protein truncation/degradation happened. Though we also examined the effect of further proteinase inhibitor addition on the purification steps, such 2E protein truncation/degradation still happened (not shown). Although thrombin treatment produced 2E protein, the efficiency and yield were not satisfying to obtain the full-length 2E protein (**Fig. R2b**). While we have successfully expressed and purified other recombinant proteins in bacteria (Bekdash *et al. Cell Rep. Methods* 2021, PMID: 35475001), for this manuscript revision, unfortunately, we could not prepare the purified recombinant full-length 2E protein sample suitable for binding assays.

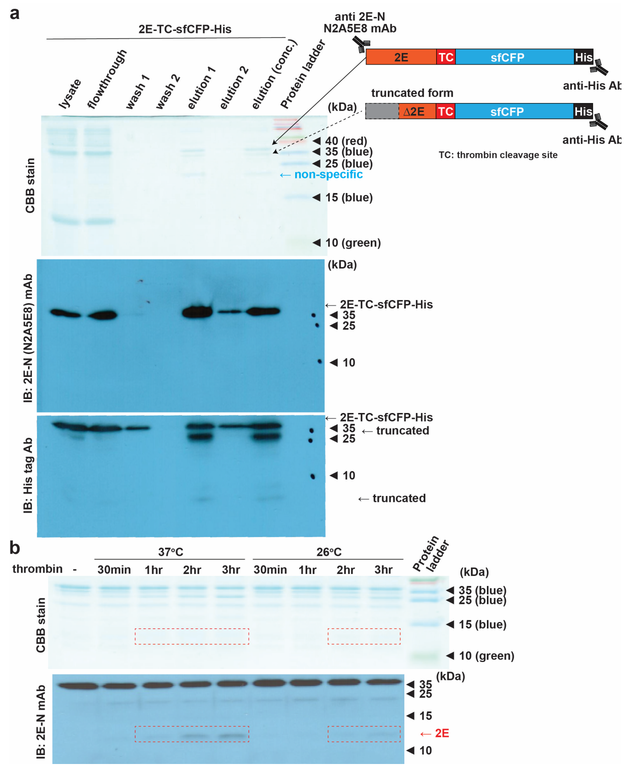

Fig. R2 | Applying thrombin cleavage site for His tag-mediated 2E protein purification. (**a**) Representative CBB stain and immunoblot images of 2E proteins with thrombin cleavage site (TC), sfCFP and His tag. (**b**) Thrombin treatment to cleave the fusion protein. Representative CBB stain and immunoblot images of the treated 2E proteins are shown.

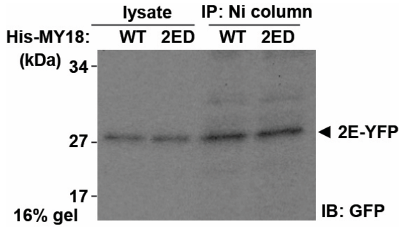

Fig. R3 | Co-immunoprecipitation of 2E protein and MY18 peptides. (a) Immunoprecipitation of 2E protein using Ni column and HEK 293T cells co-transfected with 2E-YFP and 6xHis-MY18-2ED or -WT constructs. For co-transfection, the ration of 2E-YFP to His-MY18 was 1:1.

Alternatively, we conducted co-immunoprecipitation using His-tagged MY18 wild-type and 2ED mutant peptides to pull down the full-length 2E proteins using mammalian heterologous expression system, although it'd be not as quantitative as SPR/BLI. The results demonstrate there was no obvious difference in 2E protein pull-down efficiency between the wild-type and 2ED mutant peptides (**Fig. R3**), though we tested two different con-transfection ratios (2E-YFP: His-MY18, 1:1 and 1:3, not shown), while ELISA demonstrates that there was significant difference in the affinity to anti 2E-N N2A5E8 mAb between the wild-type and 2ED mutant peptides (**Fig. S2c**).

We really appreciate this reviewer's advice, which enabled us for taking various approaches to address the question. We truly thank this reviewer for providing the helpful comment and advice.

===

Minor issues, but important:

The authors use wrong citations. For example, line 82, in what way reference 11 is related to E oligomerization? The paper is a study of a small peptide at the C-terminal domain, and its interaction with another protein.

We thank this reviewer for checking the reference. We removed the reference from the sentence.

In what way reference 14, which is just a review with a minor reference to E has contributed to E oligomerization knowledge? Please cite primary literature in which observations are first reported rather than reviews, in order to give credit where credit is due. But this requires to actually read the papers in question, not just the title.

We appreciate this comment. We have revised the reference used for E protein oligomerization.

The title of the paper is odd. "Developing inhibitory peptide against SARS-CoV-2 envelope". The authors are just careless in the use of language. The envelope of a virus is the lipid membrane that surrounds it, not a protein. They should refer in the title to the envelope PROTEIN. Grammatically, it is also wrong. Either 'Developing inhibitory peptideS' or 'Developing AN inhibitory peptide' would be correct.

We appreciate this reviewer's advice. Following it, we revised the tile to *"Developing inhibitory peptides against SARS-CoV-2 Envelope protein*".

The two last paragraphs in the Discussion should be at least partially merged; they look repetitive (lines 356 and 363).

We thank this reviewer for pointing it out. Following it, we revised the discussion.

Reviewer #2 (Remarks to the Author)

The authors addressed the reviewer's comments successfully except for the followings.

(Page 7 line 184) "…demonstrating a significant reduction of viral release from iPep-SARS2-E-treated cells." Based on the result in Fig. 5e, the authors cannot tell the decrease in N gene copy number is due to the reduction in viral release. This could be due to defect in virus assembly as well. If they want to distinguish virus assembly and secretion, they might need to quantify intracellular vs. extracellular infectious particles. In addition, measuring infectious virus titers is recommended rather than the qRT-PCR for quantification.

(Author response) We thank the reviewer for this advice. Following it, we have conducted further experiments to examine whether intracellular particles are infectious (Fig. S6b,c). The result suggests that the intracellular particles are still infectious. We really appreciate this reviewer's advice to improve our manuscript.

The authors addressed infectivity of intracellular particles by washing and trypsinization. However, it is questionable whether this procedure would release intracellular infectious particles successfully. Multiple freeze-thaw cycles would be a better method for releasing these intracellular particles.

We apologize to the reviewer that the experimental design was not stated clearly. As shown in the revised Fig. S6b, post viral infection at 0.1 MOI overnight, infected cells were trypsinized and washed extensively with PBS to remove free infectious viral particles. The cells were then counted to check viability before replating onto 4x 10^4 uninfected fresh Vero-E6 cells (seeded the night before) and incubated at 37°C/5%CO_2. Cytopathicity/infectivity developed from this incubation were scored 72 hours post replating and incubation. This protocol of assessing the infectivity of intracellular infectious virus was similar to the commonly used end point titration methods of co-culturing donor infected cells with target cells:

(ref) Kruglova, N. *et al.* 2021 *Viruses.* (https://www.mdpi.com/1999-4915/13/6/1133)

Nair, M.S. *et al.* 2023 *J. Ethnopharmacology.* (https://doi.org/10.1016/j.jep.2023.116291)

Thompson, R.E. *et al.* 2023 *J. Virol.* (https://doi.org/10.1128/jvi.00030-23)

Freeze-thaw cycles of the infected cells suggested by the reviewer would be a reasonable method to release viruses for assessing the infectivity of cell-free virus infection. However, it would not allow us to examine the cell-to-cell virus contact mediated infection. Since newly produced beta coronaviruses are known to release from the vesicles via lysosomes rather than secretory biosynthetic pathway (Ghosh, S. *et al.* 2020 *Cell*. https://doi.org/10.1016/j.cell.2020.10.039), our experimental strategy would enable for infectious viral particles released from the infected producing cells and give a chance to establish a new round of infection in uninfected cells via both cell-free and cell-to-cell infection. Even in such a stringent assay condition, there was a significant, albeit modest reduction of cytopathic effect in post-iPep-SARS2-E treatment as compared to untreated virus control (Fig. S6c), demonstrating that iPep-SARS2-E reduces viral cytotoxicity, but the intracellular virus is still infectious. We appreciate this reviewer's comment to revise and improve our manuscript.

(Page 11 line 288) "On the other hand, we found that there is no difference in SARS-CoV-2 nucleocapsid expression (Fig. 5d), suggesting no effect of iPep-SARS2-E on the viral entry." This statement needs more experimental evidences. For example, the authors need to conduct experiments with pseudovirus although in this case most of pseudovirus systems do not contain E protein.

(Author response) We appreciate this comment. Following it, we have conducted a new experiment using a pseudo virus containing E, M and S with YFP reporter to examine the effect of iPep-SARS2-E on the virus entry. We found no significant effect of iPep-SARS2-E on virus entry (Fig. R3/Fig. 8e,f). Following the new result, we have updated our discussion to explain the molecular mechanism underlying the effect of iPep-SARS2-E on SARS-CoV-2 function.

A pseudovirus containing SARS-CoV-2 S, M, E proteins was produced but no data was presented concerning the characterization of this pseudovirus. The evidence of presence and functionality of S, M, E proteins should be presented. Or the authors could cite any previous report in which this pseudovirus containing SARS-CoV-2 S, M, E proteins was described.

We thank this reviewer for pointing it out. Following it, we added a related literature of the pseudo virus containing SARS-CoV-2 Spike, M and E proteins (Wang *et al. Int. J. Mol. Sci.* 2021. PMC8396568).
